# The Role of Ultrasound Across the Inflammatory Arthritis Continuum: Focus on “At-Risk” Individuals

**DOI:** 10.3389/fmed.2020.587827

**Published:** 2020-10-30

**Authors:** Laurence Duquenne, Rahaymin Chowdhury, Kulveer Mankia, Paul Emery

**Affiliations:** ^1^Leeds Biomedical Research Centre—NIHR, Leeds, United Kingdom; ^2^Leeds Institute of Rheumatic and Musculoskeletal Medicine, University of Leeds, Leeds, United Kingdom; ^3^Leeds Teaching Hospital Trust, Leeds, United Kingdom

**Keywords:** arthritis (including rheumatoid arthritis), ultrasound, at risk, prediction, ACPA, anti-cyclic citrullinated peptide antibodies

## Abstract

In individuals at-risk of developing inflammatory arthritis, the value of an ultrasound (US) scan assessment to predict progression has been demonstrated repeatedly. However, depending on recruitment criteria, these individuals may be at different stages in the arthritis development continuum, therefore representing a heterogeneous population. As a consequence, the predictive value of ultrasound results may differ between cohorts. As other reviews have focused on the challenges in population recruitment or have combined biomarkers predicting value according to one recruitment pathway, we wanted to focus on the sole use of ultrasound assessment and its variation according to population recruitment criteria. In this review, we discuss the use of ultrasound in the different at-risk populations across the inflammatory arthritis disease continuum. This review demonstrates that although some sub-population data is scarce, ultrasound is best predictive in three at-risk populations: those with a positive ACPA test in the context of non-specific MSK symptoms, those with clinically suspect arthralgia and those with palindromic rheumatism. We consider that ultrasound assessment will be a cornerstone in prediction risk modeling and prevention studies of the preclinical phases of IA in the future.

## Introduction

Since the availability of biologic therapies, rheumatology practice has entered a new era where achieving damage free remission in rheumatoid arthritis (RA) is not only feasible, but common. As early treatment decreases long term joint damage and impaired quality of life, much effort has been made to refer, diagnose and treat patients early (1). We are now evolving toward the next stage of inflammatory arthritis management: prevention. The new priority is to identify individuals that might eventually develop inflammatory arthritis (IA): the “At-risk” individuals and treat them before arthritis occurs thereby preventing progression to RA. At-risk individuals with subclinical inflammation are at increased risk of arthritis development. It is therefore logical to suggest that this population should be considered for treatment, therefore should be included in the “window of opportunity” (2).

One of the many challenges is that arthritis development is a late step in a long process sometimes called “the inflammatory arthritis disease continuum” (3), where the preclinical phase includes, genetic, environmental, and systemic factors which may arise years before arthritis occurs (4, 5). When recruited into research cohorts, at-risk individuals might be at different stages of the continuum therefore representing heterogeneous populations, with differing risks of progression to IA.

**Figure 1:**
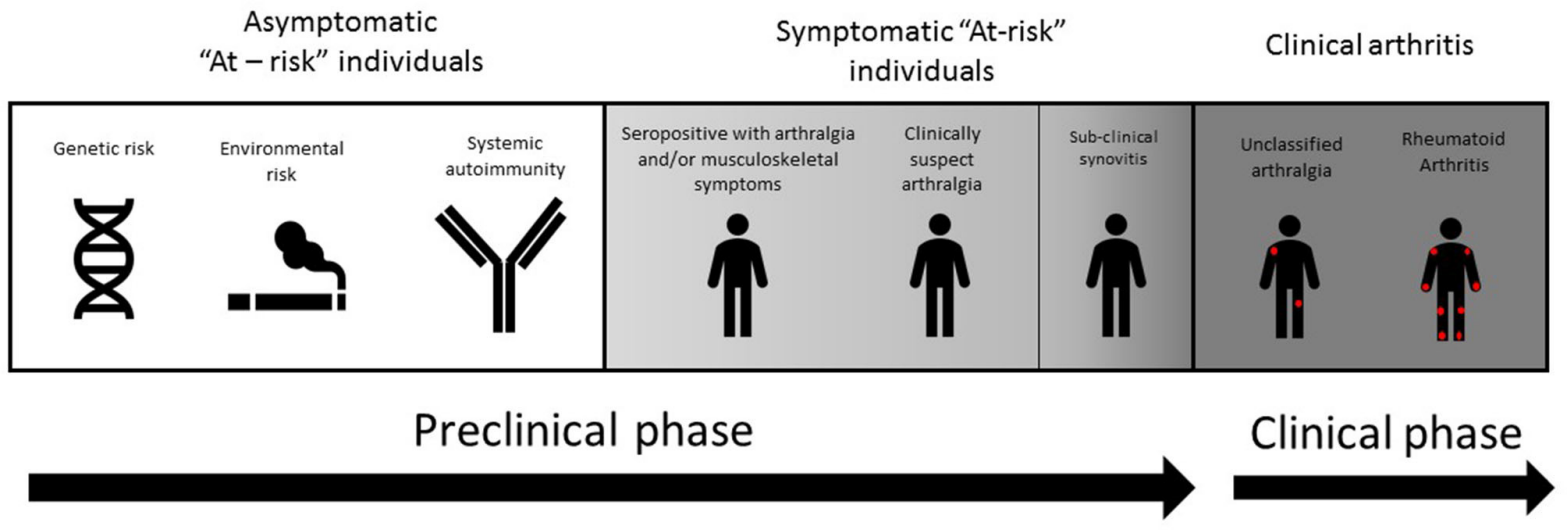
Evolution toward rheumatoid arthritis (RA) can be considered as a disease continuum encompassing pathogenic phases which conclude in the development of arthritis. Preclinical phases can comprise of a symptomatic at-risk individuals with genetic risk (e.g. an affected first degree relative), environmental risk (including smoking and mucosal inflammation), and RA-related systematic autoimmunity (e.g. ACPA antibodies). Subclinical synovitis is more often found in symptomatic at-risk individuals, either in the presence of RA associated autoantibodies, or clinically suspect arthralgia (CSA).

In this review, we discuss the use of ultrasound (US) in the different at-risk populations across the arthritis disease continuum (see Figure 1). The populations in which US may be most informative will be discussed. We included peer-reviewed, published research including, retrospective and prospective analyses, observational, and interventional studies that were relevant for research and clinical practice. Only articles in English language were included.

### US Findings in Healthy Subjects

As the aim of this review is to describe the evidence of US abnormalities in the preclinical phase of IA, it seems important to first consider their prevalence in healthy subjects. A first analysis found a power Doppler (PD) signal in 11% of hands and wrists of 27 healthy volunteers, especially in the wrists (6). Using the OMERACT consensus, analysis of 127 healthy controls (HC) matched with another 127 patients with early arthritis from the ESPOIR cohort found that 11% of the MCP joints 2–4 and fifth MTP joints analyzed had bone erosions (BE), 22% had synovial hypertrophy (SH) ≥ grade 1 and 9% ≥ grade 2 (7). Another study showed high prevalence of SH grade 1 in healthy subjects (15%) as well as in RA patients (56%), and no association with PD, tenderness or swelling, suggesting that only Grade ≥ 2 should be considered pathological (8). This is also suggested by a analysis on 46 young healthy subject who showed SE in almost 20% of them (9).

The most comprehensive analysis included 32 joints of 207 HC subjects (6,621 joints in total) (10), showing that the prevalence of US abnormalities was low at joint level but high at individual level 9 vs. 88%), and most commonly synovial effusion (SE) (69% of joints with an abnormality). BE were found only in first metatarso-phalangeal (MTP1) joints (*n* = 4) and always with PD. The most prevalent joints with US findings were the MTP1 joints followed by MTP joints 2 to 5, then wrists, metacarpo-phalangeal (MCP) joints (especially the third), and finally proximal inter-phalangeal (PIP) joints which were almost never involved. Grade 1 was the most commonly score found, higher grades were only found in feet (10). Grade 1 SE and SH were highly prevalent in healthy subjects, the authors suggested excluding these parameters from the OMERACT ultrasound protocols (7, 10). None of the analyses showed differences between men and women. Only one study showed significant effect of age, especially in the feet (10). While all of the above studies used semi-quantitative measurement, another analysis of 78 individuals determined quantitative measurement of the radio-carpal abnormalities with a greater chance to indicate RA (11).

Taken together the above studies suggest that the presence of low grade US abnormalities (especially grade 1 SE and SH and feet localization) are often present and therefore should not be considered pathognomonic for inflammatory arthritis. It is interesting to notice differences between each MTP, the first and fifth showing the most abnormalities and that larger joints such as elbows, ankles and shoulders have not been analyzed. Also, midfoot joints are not considered in any of the studies. The EULAR-OMERACT combined score defined synovitis as both PD and GS≥1 (GS including SH and SE), including only small joints, with no difference of scoring between joints (12).

### The Use of US in First Degree Relatives of Patients With RA

Because a family history of RA has been shown to increase the risk of developing this disease, the use of US as a prediction tool for the development of arthritis in first degree relatives (FDRs) of RA probands has been investigated.

A small study of 20 patients with RA and 25 of their FDRs was undertaken to explore the presence of abnormal US findings in FDRs without clinical arthritis (13, 14). Eight FDRs had arthralgia symptoms, but all were negative for RF and ACPA. The study confirmed the presence on US of inflammatory activity in FDRs (10/25 patients, 40%) and offers support for the use of US as a screening tool in this at-risk population. This study was limited by the small number of participants and did not present the US findings in detail.

A large prospective study investigated a cohort of 237 FDRs of RA patients (15). The population included a spread across the spectrum of RA development and was classified into four groups (three preclinical and one clinical). The first group were those “without risk factors,” meaning those negative for the shared epitope, anti-citrullinated protein antibody (ACPA) or rheumatoid factor (RF) and had no symptoms of possible RA (*n* = 45). The second group had risk factors, with either presence of one or two copies of the shared epitope or an ACPA positive test but no symptoms associated with possible RA (*n* = 38). The third group included subjects with inflammatory arthralgia, or self-reported symptoms associated with possible RA (*n* = 132) and the fourth group had features of unclassified arthritis (UA) (*n* = 58). The authors found that active US findings were associated with the presence of UA on examination but not with the earlier preclinical phases of RA development, including those who had genetic risk factors but were asymptomatic. There was no statistical significance between the US results of ACPA positive and negative FDRs, however it is worth noting that the US scores for these groups were quite low, [mean B-mode score (SD): 6.7 (3.6) vs 6.8 (3.6)], OR: 1.0 (95% CI: 0.9 to 1.1); mean Doppler score (SD): 0.8 (1.3) vs. 1.2 (1.9), OR: 1.2 [95% CI: (0.9 to 1.6]). In addition, there were no demographic or clinical risk factors significantly associated with active US findings except for older age.

These results do not support a role for US in FDRs without symptoms, as part of a screening strategy for preclinical RA detection in FDRs, with the possible exception of those with UA features. Further analysis on the individuals who are positive with the shared epitope and/or an ACPA tests would improve categorizing risk populations.

### The Use of US in Individuals With Clinically Suspect Arthralgia

Other studies have explored the use of ultrasound in predicting IA in patients with clinically suspect arthralgia (CSA). CSA was defined by EULAR as a set of characteristics to be used in patients with arthralgia without clinical arthritis and without other diagnosis or explanation for the arthralgia (16).

Although many of the following studies were performed prior to this definition, they do follow the same theme of including patients who had inflammatory arthralgia but had no clinical synovitis (CS). An example of this is a large multicentre study which included patients who presented with at least two painful joints but no CS, with symptoms that lasted less than a year (17). Of the 196 patients who were included, 159 patients completed follow up over a 1 year period, only 15% were ACPA positive. The authors defined US synovitis as GS ≥ 2 and PD ≥ 1 and reported a statistically significant association between US synovitis and prediction of IA (OR 3.03, 95% [CI: 1.69–5.41]). They concluded that the lack of US synovitis was a strong negative predictor for IA. These findings were further supported by a retrospective analysis of 80 consecutive patients using SONAR B-mode criteria to determine significant synovitis (18, 19). They found that significant US GS synovitis appeared to be the only independent predictor of RA on multivariate analysis (OR 7.4 [95%CI: 1.19–42.8]) in ACPA negative patients who presented with poly-arthralgia and no CS.

A recent study compared the US and MRI results of 70 individuals with CSA (*n* = 40) and early IA (*n* = 30). They showed an overall significant correlation between both imaging techniques regarding synovitis and tenosynovitis, especially in the MCP and wrists joints, although MRI was more sensitive. Although less frequently present in the CSA subgroup, similar results of concordance were found, but with a lower sensitivity (20).

Thus, the literature would suggest that US does add additional value to clinical and laboratory investigations in predicting IA in those with CSA. However, there are a paucity of data on US as MRI has been preferred and further US investigation is recommended.

### The Use of US in ACPA (and/or RF) Positive Individuals With Musculoskeletal Symptoms

There have been various studies on the use of US to predict RA in at-risk populations who have been selected on the base of an ACPA and/or RF positive, with MSK symptoms (including arthralgia), but no CS.

An important observational cohort study investigating US as a predictor for IA in ACPA positive at-risk individuals was conducted by a group in Leeds (21). The 100 consecutive participants included were ACPA positive, had a new non-specific musculoskeletal (MSK) symptom, but no CS. They demonstrated in multivariable analysis a significant association between PD at the patient level and the development of IA (HR 1.88 [95% CI 1.07–3.29]) and incorporated it in a prediction model including serological and clinical measures. In Amsterdam, a prospective cohort study of 192 participants who were ACPA and/or RF positive, found that Gray Scale (GS) and PD were predictive for IA at the joint level, but this did not reach statistical significance at the patient level, meaning that there was a significance in US findings predicting which joint would progress to clinical arthritis, but not which patients would progress to IA (22). It is useful to note that US protocols varied between both studies; while Amsterdam's US protocol included only painful, adjacent, and contralateral joints if hands were involved, the Leeds study included all MCP joints, PIP joints and both wrists. A follow up study in Leeds included 136 individuals and added MTP joints to the analysis (23), they concluded that all US findings (BE grade ≥ 1, GS grade ≥ 2 and PD grade ≥ 1) could predict progression to IA and its timing, with the risk being greatest in those patients with at least one joint with PD signal on US. Unlike the Amsterdam cohort, the predictive value of PD was significant at both the joint and patient level. The discrepancy in findings may have been due to the differing inclusion criteria in the studies. While the Amsterdam cohort included about a third of RF positive—ACPA negative individuals, Leeds group included only ACPA positivity, suggesting that individuals in the two cohorts could have been at different level of progression risk. Additionally, the US protocols in both studies were different, Leeds scanned 32 joints including bilateral wrists, MCPs, PIPs and MTPs. Both studies were amongst the first to include only patients without CS.

A follow up prospective cohort study from Amsterdam (24) included a cohort of 163 seropositive (RF and/or ACPA) patients who had arthralgia but no CS in a prospective cohort. After excluding metatarsophalangeal joints (MTPs), they showed that GS had a significant predictive value to progression to IA (OR 6.6 [95%CI 1.9–22]). Unlike the other above studies, PD was not found to be predictive in this study. The authors attributed this main difference to the alternative scanning protocol and technical differences in US machines. Differences can also be ascribed to the inclusion criteria, as Nam et al. included ACPA positive patients only (18).

In two further analyses, the first (25) analyzed US images from 319 patients and found that the number of joints with PD or tenosynovitis (TSV) was predictive of progression to IA with high specificity and moderate sensitivity with respective hazard ratios of 1.2 (*p* = 0.026) and 1.13 (*p* = 0.025), the addition of ACPA titer improved the predictive value of the number of joints with PD with a specificity/sensitivity of 0.92/0.34 (AUC 0.964). The authors also suggested that a selection of joints—mainly in the hands and feet—with better predictive power could improve US sensitivity. Another multivariable analysis on the same population (*n* = 488) showed that individuals with 1–3 joints with a PD signal or 1–2 with BE were twice as likely to develop IA, those with ≥ 4 joints with a PD signal were more than six times more likely (26). A more recent study (27) analyzed baseline US scans of a further 419 CCP positive at-risk individuals from the Leeds CCP cohort. In this analysis, the most predictive features for the development of clinical arthritis on US were BE in >1 joint or BE combined with synovitis in the MTP5 joint (OR 10.6 [95% CI 1.9 to 60.4] *p* < 0.01) and 5.1 [95% CI 1.4 to 18.9] *p* = 0.02] respectively. While presence of BE in any joint was previously described predictive of progression to IA (23), this study suggests that some joints might be more specific for progression.

Overall, the discrepancies in some of the findings are likely due to population variability: whether selected individuals are tested positive for RF and/or ACPA, associated symptoms and set of joints analyzed, but also the factors around how to perform the US itself, defining US synovitis, the optimum number and specific joints to be incorporated in the US protocol and the use of different scoring systems.

The above studies demonstrate that there is strong evidence that all US features including GS, BE, TSV, and especially PD presence, have an important part to play in predicting IA in seropositive at-risk individuals with MSK symptoms. They also discussed scanning protocols, while a limited, focused joint set can be used to identify erosions and predict arthritis; pragmatic scanning protocol could be easily incorporated into clinical practice. Ideally future studies should use standardized US protocols and scoring systems.

### The Use of US in Individuals With Palindromic Rheumatism

Some individuals—ACPA positive or not—present with intermittent inflammatory flares, alternating between short attacks of pain and swelling and asymptomatic periods. Specific clinical and US features found in these individuals suggest that palindromic rheumatism (PR) could be a discrete pathological entity (28). However, shared risk factors with RA suggest PR could also be considered a phase of the RA continuum (3). Different studies have focused on this population, whether it be during or in-between flares of joint symptoms. In all these analyses, the proportion of ACPA positive patients ranges between 13 and 66.7%.

In 2014, a group analyzed 11 joints in the hands of 54 patients outside of a flare (29). The joints reported to be involved in the first flare varied according to ACPA status, with an increased ratio of small joints involvement in ACPA positive participants. This antibody based discrepancy was confirmed on US performed between flares. At the joint level, they found that only 2.8% of the 1,188 joints analyzed had SH≥2, with PD≥1 in 1.4% of these, mostly wrists and MCP. At the patient level, 25.9% of them presented US synovitis in at least one joint (SH≥2, or PD≥1). Patients in this cohort had long disease duration (mean duration of 11.6 years) and 61.1% were on DMARDs (mainly hydroxychloroquine). Ten patients of this study were also assessed during a flare, none of them showed periarticular US abnormalities, seven showed an intra-articular PD signal.

In a small cohort (*n* = 15), analysis of US scans taken during flares in the hands and wrists showed US synovitis in 60% of individuals (*n* = 9/15), with PD in 6 of them (30). The largest analysis to date was based on 84 PR patients during a flare (31). While 78% of the participants had signs of PD presence, only 31% of these were intra-articular PD, the rest were features of TSV and/or periarticular soft tissue inflammation. Moreover, Intra-articular PD presence and ACPA positivity were both recognized as predictors of progression to RA (respectively: OR = 2.28 [95%CI: 0.67–7.68] and OR = 6.18 [95% CI: 1.50–25.52]).

The most recent imaging analysis in PR was performed on 79 individuals comparing US and MRI (32). This analysis was the first to include treatment-naïve individuals, with a short duration of symptoms. The authors compared US examinations taken during and between flares, and showed US of the flaring area showed significantly more TSV (23 vs. 4%), more signs of extra-capsular inflammation (61 vs. 15%), particularly periarticular inflammation (39 vs. 4%), (and non-significantly different PD synovitis [23 vs. 7%]) than in-between flares. Interestingly, there was no influence of the antibody status on the US features. These results suggest that palindromic rheumatism has a discrete imaging phenotype and that some features such as ACPA positivity and intra-articular inflammation during a flare may increase the risk of developing RA.

These studies all included individuals with PR, which US has revealed some specific features such as higher proportion of extra-capsular abnormalities compared to at-risk and RA individuals. Discrepancies can be explained by recruitment criteria differences such as the use of previous medication, proportion of ACPA positive individuals and symptoms duration. Two analyses showed predominance of US extra-articular inflammation while all showed presence of sub-clinical inflammation.

### Ultrasound in Undifferentiated Arthritis

Early diagnosis is a priority, it is therefore essential to improve diagnostic success rates at first referral. Because of its power and accessibility, US is now recognized as an essential tool for IA diagnosis and management. We have discussed US findings and implication in individuals with no CS (the at-risk populations), individuals with intermittent CS (palindromic rheumatism). Here we discuss how US use has been a major tool in assessing patients at CS onset. This chapter will not focus on RA patients but those with undifferentiated arthritis (UA) who are presenting with early CS but do not meet classification criteria for a specific rheumatic disease.”

Many studies have shown US superiority to clinical examination in detecting synovitis (33–35). In a cohort of 50 ACPA and RF negative patients with UA from early arthritis clinics, they showed that IA probability increased from 6 to 8–85% depending on which two US features were present (GS = 3, PD ≥ 1 or BE presence) (36). This was confirmed in a large study (*n* = 831) where 31% of patients progressed to persistent IA, baseline serological and clinical biomarkers were already predictive of progression, but US improved all predictive values, particularly in the seronegative patients (AUC increase of 9%, *p* < 0.001) (37). Another study focused on EULAR classification criteria for RA, with the same recruitment criteria (*n* = 109), 61% of participants presented with a swollen joint, 30% were ACPA+ (compared to 15.4% in the previous study). They showed that GS≥1 improved the sensitivity of the 2010 criteria from 58 up to 78% without decreasing specificity (AUC 0.868), which was 93.7% if GS ≥ 2 or PD ≥ 1 were present, but at the price of decreased specificity which went down to 56.1% (AUC 0.844) (38). US was also shown to be especially useful in RA diagnosis in CCP negative very early UA. Indeed, in a study recruiting only CCP negative individuals with suspected UA, US synovitis significantly improved the sensitivity of the 2010 classification criteria up to 86.2% (39), meaning that US could counterbalance the absence of specific serology findings. While using a probabilistic approach depending on the practitioner's impression, another group showed that the addition of US to routine investigations increased the diagnostic certainty of UA from 31.1 to 61.2% (*p* < 0.001) (40). It is interesting that in these studies, more than half of patients presented abnormalities on first US but not all developed a persistent disease, suggesting that, focusing on the most specific US features such as PD presence and/or GS ≥ 2 might improve US accuracy. Another study showed that GS was more effective at showing synovitis than clinical examination, laboratory investigation (*p* = 0.00015), and plain film radiography (*p* = 0.0002) (41). In a study recruiting individuals with at least one swollen joint, the same improvement of the AUC for RA diagnosis was found, they also showed that MCP joints were highly specific for IA (42). Others found that selecting PD grade 2 increased discriminative ability (43).

Interestingly, in a large (*n* = 379) retrospective analysis on a cohort of patients referred to early arthritis clinic and followed for minimum 12 months, US parameters did not show significant predictive value for persistent IA in comparison to clinical parameters alone, (AUC curve both metrics: 0.91; [95% CI 0.89–0.94] [95% CI 0.88 to 0.94] respectively) (44). This same group did a further study with different scanning protocol and comparison methodology between clinical and US variables, which did show improvement of predictive values when comparing to clinical parameters alone (37).

The same team developed two “risk metrics” computerized tools using logistic regression to predict the development of persistent IA whereas the first models used multivariate and ROC curve analysis to identify discriminators of IA and the added value of US parameters (44). Another diagnostic model for progression from UA to RA was designed combining symptoms and morning stiffness durations, raised inflammatory markers, CCP and or RF positivity and PDUS presence in 1, 2 or ≥3 joints, which provided an impressive AUC of 0.919 (*n* = 149) (45).

After a RA diagnosis has been made, US has also shown a good correlation with composite scores of disease activity at all points of disease evolution, as well as with radiological damage (33, 46–48). It is suggested for use in the assessment of remission, prediction of flares, and to assess risk of relapse when tapering treatment as well as to inform the need to intensify treatment (49–55). PROMPT trial randomized 110 UA individuals to receive methotrexate (MTX) vs. placebo for 1 year depending on US results. At 5 years, they showed no difference in progression rates to RA, only a delay in progression in the treated ACPA positive participants (56).

Overall, US has proved its place in diagnosis, disease activity and remission assessment while, for now, US driven trials have shown variable results.

## Discussion

The main challenge in populations at-risk of RA is to categorize biomarkers that are specific, sensitive, and reproducible in predicting disease progression. Some of them may be present years before progression and remain stable—such as specific antibodies or genetic predisposition—whilst others may vary with time or only appear closer to the clinical phase of the disease such as abnormalities on high-resolution imaging, for example, US. Defining the phase where sub-clinical inflammation on imaging appears is particularly important, as it represents the initial onset of articular inflammation and as such, the phase where clinical arthritis is imminent. Indeed, as one RCT has shown to delay the onset of RA (57), the preclinical phase of RA could be the optimal time point to initiate treatment as damages have not occurred yet, thus re-defining the window of opportunity. Delaying or even preventing the onset of RA will have major social, financial, and personal impact on patients and society.

Retrospective studies on RA patients were highly useful initially to find out biomarkers that were present before symptoms, but these are limited in the quantity and quality of the data. For example, not all analysis was possible on frozen blood samples, no imaging was performed, and clinical data were absent. Therefore, observational studies on RA prediction should now be all prospective. Another important challenge in at-risk population studies are the discrepancies between and within cohorts depending on the recruitment criteria. This implies that results are difficult to replicate and/or compare between populations. We discussed above the various pathways of prospective recruitment, for example, CSA and ACPA+ individuals, with a usual progression to IA rate of around one third of participants. This low progression rate increases duration of follow-up needed, tests and visits to be repeated, and the need for large cohorts to get significant results. Only a few centers are able to support this.

Identifying individuals who would benefit the most from an US assessment is of major importance. Indeed, although US sub-clinical inflammation can be found throughout the whole disease continuum toward RA, US abnormalities have shown—at present—only of predictive value for disease development in specific populations. The most representative are symptomatic at-risk individuals who have been identified by antibody positivity while in some populations, for example, in FDRs and CSA individuals, data are sparse and would benefit from further study. Another limitation is that not all analysis used the same US protocols. Even if we nowadays tend to follow EULAR/OMERACT recommendation, this has not always been the case. Although some US protocols focusing on specific joint sets are suggested in RA to improve US pragmatic use in clinic (58, 59), no joint based analysis have been performed on at-risk individuals yet. Nevertheless, focus is often on the small joints such as MCP, PIP, MTP, and wrists.

The studies investigating the role of US in FDRs do not support its use in those without symptoms. There is however a paucity of data in this area and further exploration is needed. On the other hand, the predictive value of US for IA development was greater in the individuals with MSK symptoms identified by a positive ACPA test. In this group particularly, depending on the US scan protocols and the recruitment criteria, US features have shown significant predictive value at the joint and patient level, for GS, BE, TSV, and especially for PD presence. This is more consistent if we consider the studies of individuals with more stringent inclusion criteria, with cohorts more likely to be at imminent risk of progression. In individuals with CSA, MRI-US comparison has shown good correlation, mainly for specificity. At diagnosis of early UA, some studies showed US superiority to clinical examination, efficacy in RA diagnosis, disease activity assessment, and treatment efficacy. In this population, efficacy is not dependent on the serological results and might be of more value in the seronegative individuals. It has good discriminative value, improving the classification criteria's sensitivity. In established RA, although it has been shown to be a good predictor in treatment response, remission assessment, and flare prediction, two trials comparing conventional T2T approach with US lead approach did not show significant differences in DAS28 remission, and lead to an increased treatment regimen is the US groups (60, 61). Nevertheless, secondary analysis showed that Boolean remission was more often reached in the US arm (61).

At present without guidance, rheumatologists have different approaches to managing at-risk individuals. A survey conducted in 2019 across the UK showed that 73% of practitioners would treat ACPA positive individuals if at least one joint showed PD presence on US (62). This reflects the pragmatic approach used due to the lack evidence on which treatment is the most appropriate, which population would respond well and what is the most appropriate timing to start treatment. This lack of global consensus emphasizes the need for research studies to assess these questions (63). Although individuals followed in preventive observational cohorts showed milder disease activity at progression (64), long term impact of prevention clinics have not been assessed yet. A few randomized controlled trials have been designed on individuals without CS. While one used the presence of US inflammation as part of the recruitment criteria (57), another one collected US data along the study for secondary analysis, results are not published yet (65), none of the others included US as an outcome or collected longitudinal data (66–68). Some RA treatments have been tested on individuals with UA (56) or even before CS occurs (65), the complexity here is to define the optimal high risk individuals who may benefit from treatment as well as the participant acceptance for a medication without confirmed disease (69, 70). At present, no formal economic analyses for use of US or treatment in at-risk individuals have been conducted, it therefore represents an important area for future work.

All aspects of US findings throughout the RA continuum have shown its high predictive value for progression to clinical synovitis, perhaps with PD standing out to be most predictive. However, it is difficult to compare these aspects due to the different definitions of US synovitis and scanning protocols through the studies. Overall, US does offer clear assistance in identifying sub-clinical inflammation in individuals at-risk of IA. However, we have to consider the time and resources needed for systemic prevention to be put in place. All populations considered, it appears that the greatest impact on IA prediction of US examination can be found in three at-risk populations: those with a positive ACPA test in the context of non-specific MSK symptom, those with CSA, and those with palindromic rheumatism. Since it has shown such good predictive value in IA and in the preclinical phases of IA, it is expected that US will be a cornerstone in prediction risk modeling and prevention studies. Nonetheless, further studies with unified selection criteria, specific joints and/or feature selections are still needed to improve US impact relevance.

## Author Contributions

LD and RC have participated with equal contribution and would like to share first authorship. KM: important part in topic choices, discussion, proof reading, and time input. PE: topic choices, discussion, and final approval for submission. All authors contributed to the article and approved the submitted version.

## Conflict of Interest

KM reports personal fees from Abbvie, UCB, and Eli Lilly, outside the submitted work and research grants from BMS, Eli Lilly. PE reports consultant fees from BMS, AbbVie, Gilead, Galapagos, Lilly, MSD, Pfizer, Novartis, Roche, Samsung outside the submitted work and research grants from UCB, AbbVie, Lilly, Novartis, BMS, Pfizer, MSD, and Roche, outside the submitted work. The remaining authors declare that the research was conducted in the absence of any commercial or financial relationships that could be construed as a potential conflict of interest.
